# Basic Life Support (BLS) Knowledge Among the Ha’il Region Population, Saudi Arabia: A Cross-Sectional Study

**DOI:** 10.7759/cureus.75427

**Published:** 2024-12-09

**Authors:** Layla H Alenzi, Bodor Z Alshammari, Atheer Alghurayes, Noor Alharthi, Albandri M Alghris, Falah Alshammari

**Affiliations:** 1 Dentistry, College of Dentistry, University of Ha'il, Ha'il, SAU; 2 Dental Public Health, College of Dentistry, University of Ha'il, Ha'il, SAU

**Keywords:** basic life support, general public, ha’il, knowledge, saudi arabia

## Abstract

Aims and background

This study aims to evaluate Basic Life Support (BLS) knowledge among residents of the Ha’il region. It will reveal the public's familiarity with BLS and identify sources of BLS knowledge. The findings can guide policymakers in improving BLS training, potentially saving lives, especially during cardiac emergencies. Annually, heart attacks and strokes cause millions of deaths, with projections indicating a potential increase without intervention. In Saudi Arabia and globally, public awareness and education on BLS are insufficient, highlighting the need for better dissemination of BLS knowledge to improve emergency response before ambulance arrival.

Objectives

Measuring the level of BLS knowledge among the Ha’il region population, seeking the sources from which individuals acquire their BLS knowledge, and determining various factors that may influence BLS knowledge among the Ha’il population.

Methods

In Ha’il, Saudi Arabia, the survey was disseminated to the general public and included 380 participants. All participants are residents of Ha’il and are at least 18 years old. Data were collected via an online questionnaire, which was validated and translated into Arabic.

Results

The study surveyed 380 adults aged 18-36, with 62% (n = 236) females and 38% (n = 144) males. Most (60%) had a university degree, 46% were employed, and 29% were students. Slightly over half (51%) of participants were aware of BLS. Among those aware, 17% were very aware of the importance of BLS, while 42% had moderate awareness. The remaining participants had no awareness of BLS. A significant majority (83%) of participants believed that BLS increases the chances of patient survival.

Conclusions

The vast majority of people think that BLS is essential, but awareness and training are lacking. There were gender disparities in BLS willingness, with women being more conscious of and inclined to pursue BLS training. Furthermore, age and BLS awareness were related.

## Introduction

Basic Life Support (BLS) is a prehospital medical care procedure designed to help maintain a patient's vital functions until they can be transported to appropriate medical care [1,2]. The primary goal of BLS is to sustain the heart’s ability to pump blood, ensuring that oxygen reaches the brain and vital tissues, thus maintaining their viability until more extensive medical treatment can be administered [2]. Some medical conditions and injuries can be managed effectively with BLS alone, reducing the need for professional medical intervention [3,4]. This underscores the importance of widespread BLS knowledge and training, which can significantly enhance patient survival rates. Moreover, given that heart attacks are a leading cause of death globally, BLS is especially critical in scenarios where immediate medical treatment is unavailable [5].

Recent systematic reviews have assessed the level of BLS knowledge among the Arab population in nine countries, revealing that non-medical students generally have above-average knowledge. However, Saudi Arabia was not included in this study. In Saudi Arabia, a study conducted in Jeddah among non-medical students found that only 39% had knowledge of BLS [6]. Multiple studies have measured BLS knowledge in Saudi Arabia among healthcare students and school teachers [7-9]. A study in Riyadh involving female university students from five health colleges indicated poor BLS knowledge but a high willingness to learn [7]. Another study in Riyadh targeting dental students found that students in implants and oral surgery tend to have knowledge of BLS [10]. Another study targeted dental students in northern Saudi Arabia and found very good information among the students regarding BLS, especially male students [10-12]. In Jizan, research among university students showed that both male and female students had average BLS knowledge, with male students displaying a greater willingness to train [13]. Furthermore, a study targeting health students reported that 83% wanted BLS training to be integrated into their university curriculum [8]. However, none of these studies have targeted the general population.

The primary aim of this study is to measure the level of BLS knowledge among the population in the Ha'il region. This research will provide valuable insights into the percentage of the population with BLS knowledge, identify the sources of BLS knowledge, and survey these sources. Such data will aid policymakers in selecting effective methods to educate and train the population in BLS, ultimately contributing to saving lives.

## Materials and methods

Study design

This study employed a cross-sectional study design, utilizing quantitative methods through a questionnaire survey.

Inclusion and exclusion criteria

The participants of the study followed an inclusion criterion, which includes any male or female adults aged 18 years and above and living in Ha'il city, Saudi Arabia. Any participants who do not match the criteria are excluded from the study.

Sample size and data collection

The sample size was determined using the formula: $N = \frac{(Z_\alpha)^2 \cdot p(1 - p)}{d^2}$, considering a 5% sampling error and a 95% confidence level. The symbol 'N' represents the expected sample size; 'Zα​' corresponds to the 5% level of significance (1.95); 'd' is the level of precision (estimated at 0.05), and 'p' represents the percentage from previous studies (0.5). The estimated population of Ha’il city (327,941 individuals), as reported by the Saudi General Authority of Statistics, was taken into account, along with an anticipated response rate of 80%. Adjusting for a design effect of 1.5, the calculated sample size was 380. Random sampling methods were employed to ensure a representative sample across various demographic categories. This method was selected because it was deemed the most appropriate for this type of research. Data collection utilized online self-administered questionnaires, starting with demographic questions on participants' socioeconomic status (SES), including education level and occupation. The main questionnaires focused on BLS knowledge, awareness levels, perceived importance of BLS, and preferred timing for receiving BLS training. The questionnaires were translated into Arabic by three researchers (AB, LY, and FA) to ensure accuracy. After comparison and discussion, the best version was selected and further reviewed by a language expert (JA) to ensure clarity and accuracy. Participants provided informed consent before completing the survey. The questionnaires were distributed through various online platforms to reach the general public in Ha’il city, Saudi Arabia.

Statistical analysis

In this study, data analysis was performed using IBM SPSS Statistics for Windows, Version 28 (Released 2021; IBM Corp., Armonk, NY, USA). Initially, all collected data were organized in an Excel file (Microsoft® Corp., Redmond, WA, USA) and subsequently imported into SPSS for further analysis. Frequency and percentage distributions of questionnaire elements regarding BLS were computed using SPSS. Gender differences in BLS knowledge were examined using independent samples t-tests, assuming a normal distribution of scores. Differences in BLS knowledge between age groups were assessed using the Mann-Whitney U test, due to the non-normal distribution of scores. Assumptions of normality and variance homogeneity were verified using appropriate statistical tests, with a significance level set at p < 0.05.

## Results

In this current study, we included 380 participants, all of whom were adults aged between 18 and 36 years old. Over half of the participants were females (62%, or n = 236), while the remaining were males (38%, or n = 144). Regarding educational attainment, 60% of participants held a university degree, while 32% graduated from high school. The rest attended either intermediate school (6%) or elementary school (1%). Among the employed participants, 46% were employed, and 12% worked in the healthcare sector. Additionally, 29% were students, and the remaining 30% were not employed (Table 1).

**Table 1:** Summary of participants’ socio-demographic characteristics

Characteristics	Variable	Count	Percentage
Gender	Male	144	38%
	Female	236	62%
Age group	18 to 24 years old	377	99%
	25 to 36 years old	3	1%
Education level	University	230	60%
	Highschool	124	32%
	Intermediate school	22	6%
	Elementary school	4	1%
Occupation	Employees in the health sector	44	12%
	Employees in the non-health sector	109	29%
	Students	111	29%
	Unemployed	116	30%

**Figure 1:**
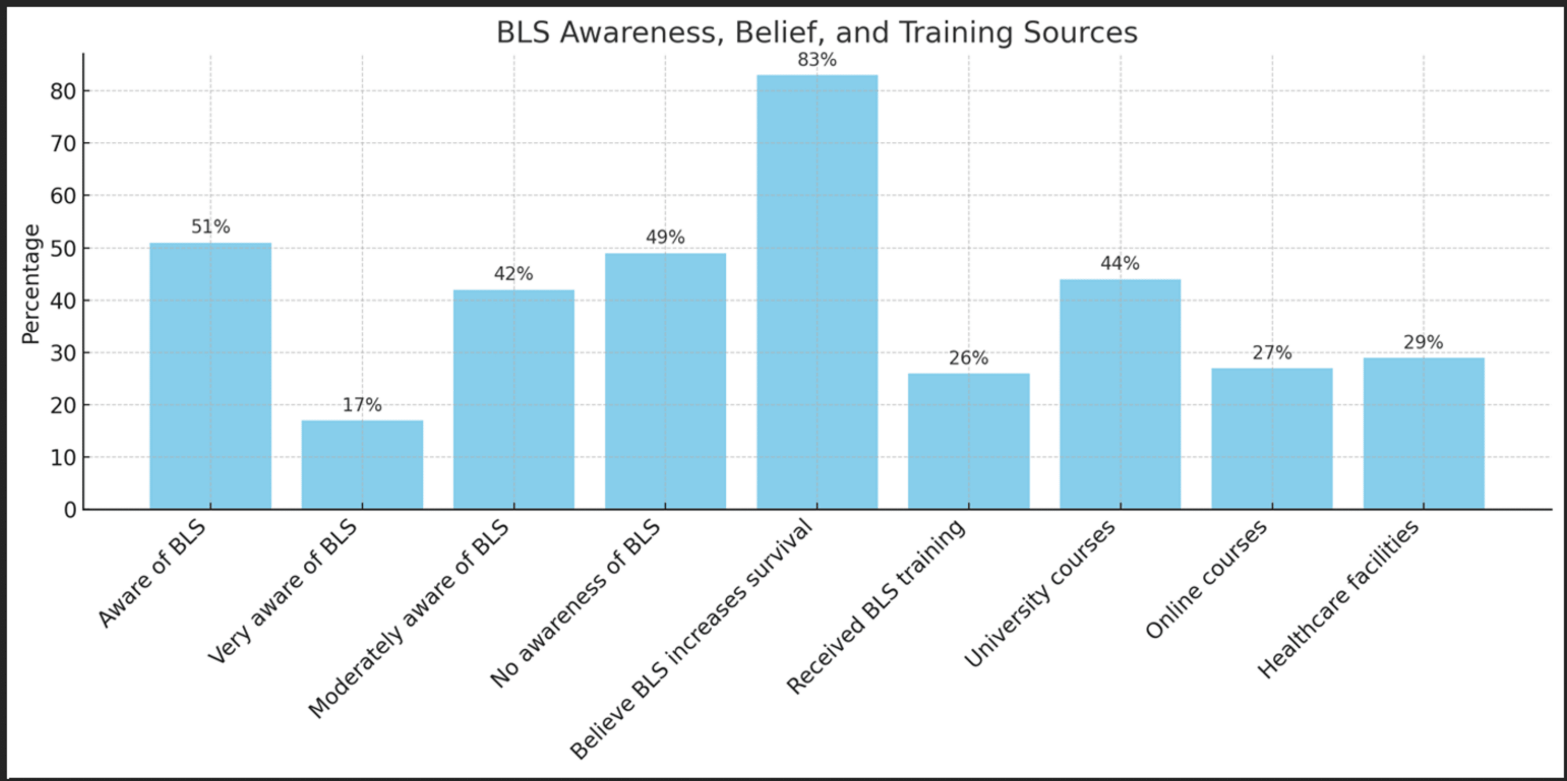
Comparison between hearing about BLS, receiving BLS training, and believing in the effectiveness of BLS in saving lives In terms of BLS awareness among participants, 51% are aware of BLS. Among those aware, 17% are very aware of its importance, and 42% have moderate awareness. On the other hand, 49% of participants have no awareness of BLS. A significant majority (83%) believe that BLS increases the chances of patient survival. Additionally, 26% have received BLS training. Sources of training include: 44% from university courses, 27% from online courses, and 29% from healthcare facilities. BLS, Basic Life Support

Slightly over half (51%) of participants were aware of BLS. Among those aware, 17% were very aware of the importance of BLS, while 42% had moderate awareness. The remaining participants had no awareness of BLS. A significant majority (83%) of participants believed that BLS increases the chances of patient survival. Despite the high belief in its effectiveness, only a quarter (26%) of participants had received BLS training. Training sources included university courses (44%), online courses (27%), and healthcare facilities (29%) (Figure 1). This data highlights the critical need for broader BLS training and education programs to enhance public health outcomes. Given that a significant portion of the population acknowledges the importance of BLS yet lacks formal training, initiatives to provide accessible and comprehensive BLS education are essential. Increasing BLS training opportunities could substantially improve emergency response outcomes, potentially saving more lives.

More than half (51%) of participants indicated they would call an ambulance in case of witnessing an accident. However, 37% chose to check the person's pulse and attempt to help the individual involved in the accident. Regarding participants' opinions on BLS education, over half (56%) of participants believed that BLS education should be included in high school curriculums, while nearly a third (29%) suggested it should be integrated into university education (Table 2).

**Table 2:** Summary of participants’ respond BLS, Basic Life Support

Variable	Respond	Count	Percentage
Heard of BLS	Yes	194	51%
				No	187	49%
	Can BLS save life	Yes	315	83%
No		12	3%
Do not know		53	14%
Aware of BLS	Very aware	64	17%
				Moderately aware	160	42%
	Not aware at all	156	41%			
	Received BLS training	Yes	99	26%
					No	281	74%
Action during facing an accident					Calling ambulance	194	51%
		Checking a person's pulse and attempting to help	141	37%
	Nothing	45	12%
First place provides BLS training	In high school	214	56%
	In university	111	29%
	Do not know	55	15%

Correlation factors with BLS

In this study, we found an association between gender and age regarding BLS knowledge and training. More females than males were knowledgeable and trained in BLS, and younger individuals were more knowledgeable and trained than older individuals. Among participants who had heard of BLS, 65% were female. Out of the participants who were very aware and moderately aware of BLS, 63% and 68% were female, respectively. Similarly, 63% of participants who received BLS training were female. When comparing age groups, the majority of participants (99%) who had heard of and received training in BLS were aged 18 to 24 years old. Additionally, 98% of participants who were very aware and moderately aware of BLS were in the same age group. Statistically, there were significant differences between females and males in terms of hearing about BLS (p-value = 0.004), being very aware (p-value = 0.052), being moderately aware (p-value = 0.0006), and receiving BLS training (p-value = 0.004). Regarding age, there was a significant difference (p-value = 0), which can be explained by the fact that most of the participants were between 18 and 24 years old (99%) (Table 3).

**Table 3:** Statistic comparative between male and female BLS, Basic Life Support

Category	Total participants	No. of female (%)	No. of male (%)	No. of ages 18-24 (%)	No. of ages 25-36 (%)	p-value
Heard of BLS	194	126 (65%)	68 (35%)	193 (99%)	1 (1%)	0.004
Very aware of BLS	64	40 (63%)	24 (37%)	64 (100%)	0	0.052
Moderately aware of BLS	160	109 (68%)	51 (32%)	160 (100%)	0	0.0006
Received BLS training	99	62 (63%)	37 (37%)	98 (99%)	1 (1%)	0.004

## Discussion

This research examines knowledge, awareness, and attitudes toward BLS training across diverse populations, including university students, the general public, and high school students. The aim is to evaluate current BLS education levels, identify areas for improvement, and highlight disparities. Findings from this survey indicate that approximately 51% of participants had heard of BLS, with only 17% categorized as 'very aware' and 42% as moderately aware of its relevance. In such a situation, knowledge is important for increasing the chances of lifesaving.

This study showed that a significant majority (83%) of participants believed that BLS could improve patient survival, reflecting a positive attitude toward its importance. However, only 25% of participants had received formal BLS training, revealing a gap between perceived importance and actual preparedness. Almutairi et al.'s study also emphasized the need to enhance attitudes and views toward BLS, as a sizable number of the general populace were unaware of its significance.

In this study, comparative analysis provides a comprehensive view of current BLS knowledge, awareness, and training across different groups, finding that females are more likely to learn BLS and undergo training compared with males. Furthermore, young people also show a higher tendency toward learning BLS. This was also found by Amacher et al. (2017) [14], who claimed that females show a higher interest in learning BLS. On the other hand, Shaheen et al. (2023) [6] observed higher BLS knowledge among males in Arab countries, pointing to cultural influences that warrant further investigation.

Regarding educational preferences, out of 380 participants, 56.3% advocated for BLS training in high schools, while 29.2% preferred undergraduate settings. These insights align with Iserbyt's (2016) [15] findings that early exposure to BLS education during adolescence fosters better preparedness. The rationale seems to be that teaching BLS to students at a younger age, before they become fully independent adults, would allow them to develop fluency and comfort with the skills sooner. The majority opinion favored incorporating BLS into high school curriculums. The alternative view, held by a sizable minority, was that BLS education should be reserved for the university setting, when students have matured and taken on more accountability for their own learning.

Regarding training sources, only 26% of participants received BLS training, with less than half acquiring it from healthcare settings. This emphasizes the need for integrating comprehensive BLS education into healthcare curricula, as highlighted by studies showing that medical and dental students exhibit higher BLS readiness [15-17]. Research by Spooner et al. (2007) [18] argued that effective BLS training should span at least six weeks for optimal knowledge retention and application. Additionally, this study underscores the need for enhanced BLS education initiatives to bolster community resilience and emergency response capabilities.

Participants' actions during emergencies varied: while over half indicated they would call an ambulance, 12% admitted they would not intervene, underscoring potential barriers, such as lack of knowledge. Addressing these gaps could improve public willingness to assist during emergencies. According to the Saudi Ministry of Health (MOH) [1], they have provided a call center to offer instructions that could help accident witnesses assist the suffering individual. However, the authors are unsure if the population is aware of this information. Therefore, the authors believe this kind of information should be further investigated. Furthermore, the authors suggest that these instructions should be provided by all emergency call centers, specifically ambulance call centers. As a result, it is suggested that all operators of emergency call centers, including traffic employees, receive BLS training. This training should be at least six weeks long, as suggested by a study conducted by Spooner et al. (2007) [18]. Almutairi et al. (2023) found a comparable lack of awareness among the general public in Saudi Arabia [19].

Furthermore, the limitations of the study include the small sample size and the cross-sectional methodology, which provides a snapshot of current knowledge at a certain point in time. Longitudinal studies that track changes over time would be beneficial.

## Conclusions

The study found that the majority of participants believe in the importance of BLS. However, there was a lack of awareness and training, with a sizable portion of the general populace unaware of its significance. The findings also revealed gender differences in BLS willingness, with females being more aware and more likely to train in BLS. Additionally, age was associated with BLS awareness and training willingness, as younger individuals were more likely to train in BLS compared to older individuals.

## Appendices

Do you agree to participate in this study?

Yes No

Age (Please select the appropriate age range):

18-24 25-34 35-44 45-54 55-64 60+

Gender:

Male Female 

Education:

Elementary Intermediate High school University

Occupation:

An employee in the health sector An employee in the non-health sector Student Unemployed Retired Others

Have you heard about Basic Life Support (BLS) before?

Yes, I am familiar with BLS No, I have never heard of BLS before How aware are you of the importance of BLS knowledge and skills? Very aware Moderately aware Not aware at all

Have you received any formal BLS training in the past?

Yes No

If yes, where did you receive the training?

University/College Online course Healthcare facility Other

Have you faced any challenges or barriers in acquiring BLS knowledge?

Lack of training opportunities Time constraints Lack of motivation NA

Do you think learning the basics of basic life support can make a difference in saving an injured person's life, improving the quality of trauma care and reducing mortality?

Yes No I don’t know 

Do you think that awareness of how to manage and transport patients in a pre-hospital environment may give the patient a greater chance of survival?

Yes No I don’t know 

What do you think is right if you witness a car accident on your way?

Attempt to rescue the person and check for a pulse Call for an ambulance immediately I will be waiting for a qualified person

Ambulance services receive thousands of calls from suspected cardiac arrest cases every year. However, only half of them were able to be saved at the latest as the rest could not survive by not receiving CPR, do you think that widespread awareness of the basics of life support may be a life-saving audience until the emergency medical team arrives?

Yes No I don’t know

When do you think BLS training should first be provided?

In High School Undergraduate I don't think BLS training is important I don't know
